# Should we use extracorporeal photopheresis more often? Evidence from graft-versus-host disease patients monitored with Treg as a biomarker

**DOI:** 10.2144/fsoa-2020-0107

**Published:** 2020-08-10

**Authors:** Sérgio Machado Lopes, Susana Roncon, Ana Catarina Pinho, Filipa Bordalo, Luís Antunes, Fernando Campilho, António Campos, Altamiro Costa-Pereira

**Affiliations:** 1 Department of Cellular Therapy, Instituto Português de Oncologia do Porto, 4200-072, Porto, Portugal; 2 Faculty of Medicine, University of Porto, 4200-319 Porto, Portugal; 3 Department of Bone Marrow Transplant, Instituto Português de Oncologia do Porto, 4200-072, Porto, Portugal; 4 Department of Epidemiology, Instituto Português de Oncologia do Porto, 4200-072, Porto, Portugal; 5 Center for Health Technology & Services Research (CINTESIS), Department of Community Medicine, Information & Health Decision Sciences (MEDCIDS), Faculty of Medicine, University of Porto, 4200-319, Porto, Portugal

**Keywords:** ECP, extracorporeal photopheresis, GvHD, immunotherapy, Treg

## Abstract

**Background:**

Chronic graft-versus-host disease (cGvHD) is a major complication after allogeneic hematopoietic cell transplantation. Extracorporeal photopheresis (ECP) is an immunotherapy treatment for cGvHD, although suitable response biomarkers are lacking.

**Materials & methods:**

We analyzed data from six cGvHD patients undergoing ECP at a reference center from 826 to 2866 days. Circulating Tregs were enumerated, patient’s clinical evolution, immunosuppression dose and adverse events (AEs) registered.

**Results:**

We observed an increase in Tregs, a decrease in immunosuppression dosage and symptoms improvement. Mild AEs occurred at a very low rate.

**Conclusion:**

In these patients, the improvement of cGvHD, with low AEs, confirms a place for ECP as treatment. Improvements were accompanied by an increase in circulating Tregs, suggesting their role as a biomarker.

## Lay abstract

Hematopoietic stem cell transplant is a lifesaving treatment, involving collecting blood-forming cells from a healthy donor and infusing them into a patient. Donor immune cells may attack patient tissues causing severe complications, including death (graft-versus-host disease [GvHD]). Primary treatment for GvHD are steroids. Extracorporeal photopheresis (ECP) is an alternative treatment that induces immunological tolerance, in part, due to Treg. We studied both clinical response and circulating Treg in six patients treated with ECP for up to 8 years. ECP responding patients had less GvHD, reduced the dose of steroids and increased the number of Treg.

Allogeneic hematopoietic cell transplantation (alloHSCT) is a treatment used for a variety of malignant and nonmalignant diseases of the bone marrow and immune system. Despite great improvements to the procedure, alloHSCT is often associated with serious immunological complications.

The most common, and lethal, of such complications is graft-versus-host disease (GvHD). This occurs when immune competent cells from the donor target the patient’s tissues, through complex, multiparameter mechanisms. Unlike acute GvHD, which depends mainly on an alloreactivity response of donor T cells to HLA-mismatched recipient tissues, chronic graft-versus-host disease (cGvHD) shares many features with autoimmune disorders, with involvement of the B cell compartment and antibody mediated response [1,2].

Extracorporeal photopheresis (ECP) is a cell-based immunotherapy developed in 1987, primarily for cutaneous T-cell lymphomas, but has since been used in autoimmune disorders, such as Chron’s disease, systematic lupus erythropoiesis and GvHD [3]. It involves collecting peripheral blood mononuclear cells (MNC), treating them with a photosensitizing agent (8-methoxypsolaren, 8-MOP), exposure of the treated cells to UV-A light (causing apoptosis) and reinfusion to the patient [4,5]. This procedure can be performed with a single piece of equipment (online protocol) or in a two-stage offline protocol, where MNC are collected in an apheresis equipment and later illuminated. The former has the advantage of being performed in a closed circuit, but with the latter it is easier to evaluate the quality and quantity of treated MNC, adjust the product volume in low weight patients and to further manipulate the cells if desired (such as selecting/depleting a specific cell subset before infusion).

The mechanisms of action of ECP are not fully described but include, upon clearance of apoptotic cells by antigen-presenting cells, induction of a tolerogenic phenotype with decreased stimulation of effector T cells, a shift in the cytokine profile (decrease in pro-inflammatory, such as IL-2 and IFN-γ and increase anti-inflammatory cytokines, such as IL-10 and TGF-β) and expansion of Treg [6–8]. All these mechanisms are potentially beneficial in patients with GvHD.

Although ECP induces apoptosis on the lymphocytes collected, there is no observed lymphopenia after reinfusion, and patient improvement cannot be attributed to direct elimination of alloreactive T-cell clones [9]. However, these clones are more sensitive to ECP-induced apoptosis, contributing to the specific immune response observed in ECP, with no systemic immunosuppression observed [10,11].

Despite the high number of available studies and the recommendation as a second-line treatment, the use of ECP is very uneven among countries. In Europe, there are countries with well-established networks of ECP centers, and other with more sporadic use.

Monitoring cGvHD relies mostly on the clinical evolution of patients due to the lack of reliable biomarkers, so subtle evolutions may remain unnoticed. Although some advances have been made in this field, most proposed biomarkers are not routinely performed and interpretation can be difficult [12,13]. Cellular content of the MNC collection does not seem to correlate to patient’s response to ECP. Relevance of peripheral blood hematological values vary within studies, although Treg are frequently found to be altered [14,15].

In this study we aimed to evaluate the role of ECP in the treatment of patients with cGvHD and whether monitoring of T-cell subsets, especially Treg, can be used as a biomarker for response to treatment in these patients.

## Materials & methods

### Patients

We followed six cGvHD patients who were referred to the Cellular Therapy Department of the Porto Comprehensive Cancer Centre (IPO, Porto, Portugal) for ECP. On the day of treatment, a physical check-up was performed by a physician in the apheresis facility and the procedure started by the nursing staff. Blood samples were collected before and after the ECP, in order to adjust equipment settings and monitor relevant parameters (complete blood count and basic metabolic panel). During the procedure, patients were also evaluated for blood pressure, heart rate and body temperature. On the first day of each session, an ethylenediaminetetraacetic acid blood tube was sent to the flow cytometry lab for immune monitoring.

### Photopheresis procedure

From 2008 to 2014 all ECP’s were performed on an Uvar XTS system (Therakos, PA, USA), with patients performing two sessions on consecutive days, in a 15 day or monthly interval – in accordance to cGvHD severity. In long term treatments this interval may be extended depending on the physicians recommendation. In September 2014, our center replaced this device for a Cellex (also from Therakos), with no alteration in the treatment scheme. For each session a new kit was installed and patient parameters introduced as requested. The separation and illumination (1.5 J of UV-A) of the buffy coat occurs in a closed circuit, with only the addition of the 8-MOP requiring direct action from the operator.

In 2015, a two-step procedure was introduced, dubbed ‘offline’ photopheresis, in which patient’s MNC were collected with an Optia Spectra separator (TerumoBCT, CO, USA), using the continuous MNC program. After collection the bag was sent to a grade D cleanroom, the cells were transferred to an illumination bag (Macopharma, Tourcoing, France) by a sterile connection, 3 ml of 8-MOP was added and the total volume adjusted to 300 ml with saline, if necessary. The bag was placed in a MacoGenic G2 UV-A illuminator (Macopharma) and the cells exposed to 2 J/cm^2^ of UV-A light for 15 min. Cells were then sent back to the apheresis unit and reinfused into the patient.

The initial offline protocol consisted of a single day treatment every 15 days. After sharing experience with other experts, an intensification of the protocol was adopted in April 2017, with all patients performing ECP on two consecutive days, weekly in the first month and then every 15 days.

For each patient, data was pooled and analyzed together, regardless of the ECP protocol performed. Despite existing differences in the final product, both protocols can – and usually do – substitute each other [16]. Patient five (Pt5) began ECP in 2016 and Pt6 in 2017, having performed only the offline methodology.

### Immune response monitoring

In the flow cytometry lab, patient’s blood was analyzed. T lymphocytes, T helper (Th), cytotoxic T (Tc) and Treg cells percentage were quantified by flow cytometry with the following fluorochrome-conjugated monoclonal antibodies: CD45-APC, CD8-PERCP, CD25-PerCP, CD127-PE-Cy7 (BD Biosciences, CA, USA), CD4-FitC, CD3-PE (Dako, Agilent Technologies, CA, USA).

Briefly, a 100 μl blood sample was incubated with the antibodies for 15 min, at room temperature and absence of light. Red blood cells were lysed by incubating with 2 ml of FACSLyse (BD Biosciences) for 10 min. After centrifuging for 5 min at 1500 r.p.m., the supernatant was discarded, cells resuspended in 300 μl of FACSFlow (BD Biosciences) and readily acquired on a FACSCanto II flow cytometer.

**Figure 1.**
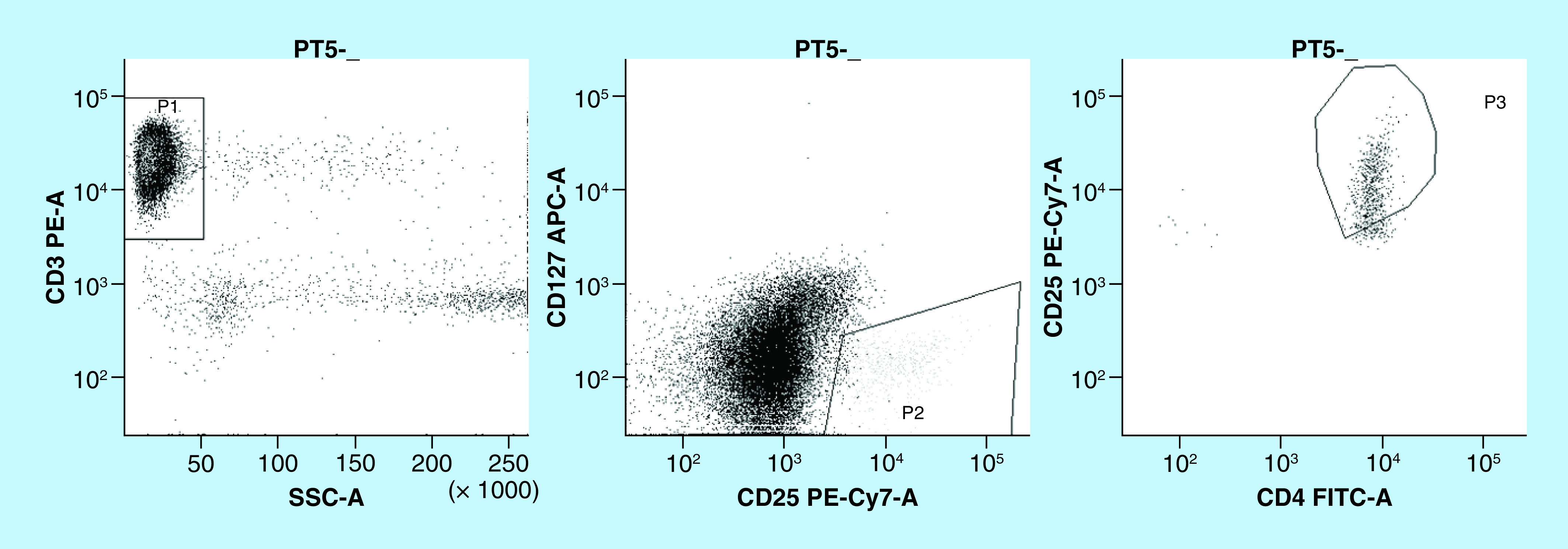
Example of flow cytometry gating strategy for Treg identification as CD3^+^, CD4^+^, CD127^-^ and CD25^++^ cells.

Total T lymphocytes were calculated as number of cells expressing the CD3 antigen. Tregs were identified as cells within the CD3^+^ population positive for the CD4 antigen, with high expression of the CD25 antigen and no expression of the CD127 antigen (Figure 1).

### Clinical data

Disease status and cGvHD severity was assessed by a physician from the Bone Marrow Transplant Unit, according to the NIH (MA, USA) criteria. In addition to monitoring patient’s vital signs, the nursing staff also registered any ECP related adverse events (AEs), in accordance to our Biovigilance System. All patient records were exported to their institutional electronic file and made accessible to IPO Porto staff.

### Data processing & statistics

Data was compiled in an excel file and statistical analysis were done using R statistical software v3.6.2. A linear mixed model with random intercept for each patient was used to evaluate the trend in Treg percentage with time.

## Results

### Patient response/evolution

Patients were treated for an average 1659 days (826–2866) from the first ECP procedure, having performed a combined total of 716 sessions (Table 1). Treatment was well tolerated in most cases, with only mild complications occurring: hematoma on the puncture site (Pts1, 2 and 3) and one syncope episode (Pt2). Worth noting that no treatment was interrupted; Pts4, 5 and 6 had no AEs reported; systemic immunosuppression or cytopenia’s were not observed (the lowest blood nucleated cell count detected was 2.5 × 10^6^/ml). Four patients stopped ECP: one after developing a carcinoma of the tongue; one due to disease stabilization; one due poor cardiac function and one with complete resolution of all symptoms. The other two are currently still on ECP program.

**Table 1.** Summary of patient’s characteristics and extracorporeal photopheresis sessions.

Case	Gender	Donor	Conditioning	GvHD prophylaxis	Time to cGvHD onset (months)	Age at first ECP	ECP duration (days)	ECPs (n)	Immune evaluations (n)
Pt1	Male	MRD	MA, BuCy	CsA + MTX	13	20	2062	162	80
Pt2	Female	MRD	RIC, FluBu	CsA + MMF	9	49	1868	91	46
Pt3	Male	MRD	RIC	CsA + MMF	120	48	1178	80	38
Pt4	Male	MRD	MA, BuCy	CsA	12	44	2866	122	68
Pt5	Female	MRD	RIC, FluBu	CsA + MMF	40	47	1155	140	79
Pt6	Male	MRD	MA, BuCy	CsA + MTX	72	59	826	121	59

BuCy: Busulfan plus cyclophosphamide; cGvHD: Chronic graft-versus-host disease; CsA: Cyclosporine A; ECP: Extracorporeal photopheresis; FluBu: Fludarabine plus busulfan; GvHD: Graft-versus-host disease; MA: Myeloablative; MMF: Mycophenolate mofetil; MRD: Matched-related donor; MTX: Methotrexate; Pt: Patient; RIC: Reduced-intensity conditioning.

Pt1 received a matched related donor (MRD) alloHSCT, for an acute lymphoblastic leukemia in first remission, in June 2008. He underwent a myeloablative conditioning with BuCy (busulfan plus cyclophosphamide), cyclosporine A (CsA) and methotrexate were used for GvHD prophylaxis. He developed grade 3 acute GvHD, with skin and gut involvement, 2 months later. He started 100 mg/day of prednisolone in October and, due to evolution of the skin lesions, CsA was added. The patient evolved to extensive cGvHD, affecting the skin and joints and began ECP in December 2009. While under the online protocol he had 2 day sessions every 15 days for 3 years, then monthly for 2 years and finally every 2 months. When the offline protocol was introduced, in March 2015, he began 1 day sessions every 2 months. He performed a total of 162 sessions until August 2015, with great improvement of the skin and only minor complications. He was able to taper prednisolone during ECP, discontinuing in 2015 and has currently reduced the CsA dose, aiming to stop all immunosuppressants.

Pt2 was diagnosed with a non-Hodgkin lymphoma in 2005 and received a MRD alloHSCT in January 2007, following a reduced-intensity conditioning (RIC) with CsA + MMF as GvHD prophylaxis. The first signs of cGvHD (skin pigmentation) appeared in October and she began CsA (400 mg/day) plus prednisolone. Symptoms progressed and MMF was added to her immunosuppression scheme in January 2009. Due to poor response, the patient was referred to our center for ECP. Treatment started in July 2009 and was suspended after 5 years with complete resolution of cGvHD symptoms, other than skin discoloration. For the first 2 years of treatment she performed 2 day sessions monthly, treatment was then reduced to every 2 months until ECP was suspended. During this period 91 sessions were performed, and she is currently disease free with no signs of cGvHD, under CsA prophylaxis (150 mg/day).

Pt3 underwent MRD alloHSCT in May 2000 for a chronic myelogenous leukemia following a RIC. He experienced disease relapse after 6 months, received a donor lymphocyte infusion and has been in complete remission (CR) since. He had a splenectomy in September 2003 as result of an autoimmune hemolytic anemia. He developed cGvHD with multiple lesions of the oral cavity and liver involvement and was referred for ECP in September 2011. Three months later he was diagnosed with a squamous-cell carcinoma (SSC) of the tongue, having a partial glossectomy in January 2012. He developed several lesions, treated with laser; in July 2013 had a SSC of the lower lip that was removed; in January 2015 a second tongue SSC, near the margin of the first surgery and underwent another partial glossectomy. ECP was temporarily suspended, having performed a total of 80 sessions, while the patient recovered from the surgery. However, his condition continued to deteriorate and in 2016 he developed yet another lesion, performed a third partial glossectomy and was definitely removed from ECP program. When treatment started, his immunosuppression scheme was 100 mg prednisolone and 300 mg CsA daily. Currently the patient is under 5 mg prednisolone every other day, 200 mg/day of CsA and 2 g of MMF. During the ECP treatment he underwent monthly 2-day sessions. This patient was considered as nonresponder to ECP, despite referring some symptom improvement.

Pt4 underwent MRD alloHSCT in August 2008 for a chronic myelogenous leukemia after a myeloablative conditioning (BuCy), with CsA + dexamethasone for GvHD prophylaxis. He developed cGvHD within a year and was treated with CsA and MMF with moderate response. Due to bad MMF tolerance he was medicated with CsA (300 mg/day) and prednisolone (60 mg/day) and proposed for ECP in March 2012. For the first 3 years of treatment he performed 2-day sessions monthly, which were then switch to every 2 months. With the offline protocol introduction in April 2015 treatment became 1-day sessions every 2 months. After 2 years the offline protocol was intensified to 2-day sessions, which is his current scheme, having performed 122 sessions. Despite maintaining extensive cGvHD with cutaneous sclerosis, the extent of the lesions and skin elasticity has been improving. In July 2017 he reduced CsA to 260 mg/day (which he maintains to date) and prednisolone to 40 mg/day; in July 2018 prednisolone was reduced to 30 mg/day and in February 2019 to 20 mg/day.

Pt5 had a MRD alloHSCT for a chronic lymphocytic leukaemia, in second remission, in November 2012, showing no sign of relapse since. She had a RIC (FluBu) and GvHD prophylaxis with CsA + MMF. A year after she developed hepatic toxicity, which was treated with prednisolone and MMF. She was diagnosed cGvHD with cutaneous sclerosis, oral mucosa, eyes and osteotendinous involvement, starting 55 mg/day prednisolone plus MMF on February 2016. The patient had steroid complications (low thyroid hormones, diabetes and cataracts) initiating ECP with the offline method in July. The first 9 months of treatment consisted of 1-day sessions, protocol was then intensified to 2-day sessions every 2 weeks, which she continues, having had 140 sessions in 1155 days. She had a good response to treatment, with improvement of all affected organs. By May 2017 prednisolone was tapered to 25 mg/day and since May 2019 to 20 mg every other day, while maintaining the MMF dose.

Pt6 was diagnosed with myelofibrosis in 2001, had his spleen removed in 2008 and developed a transfusion dependent anemia. In September 2010 he received a MRD aloHSCT, having had full intensity conditioning (BuCy). His initial GvHD prophylaxis consisted of CsA and methotrexate. While reducing the CsA dose in October 2011, he began to show symptoms of GvHD and resumed therapeutic doses of CsA in December. He remained symptom free until early 2015, displaying polyarthralgia and polyserositis with positive antinuclear antibodies, for which a dose of 70 mg/day of prednisolone was started. The patient responded well and corticoid dosage was reduced monthly to 20/10 mg on alternative days with no sign of GvHD. In 2016 he was admitted with a respiratory infection, and began to show signs of chronic kidney disease, being directed to our center for ECP. He began offline ECP in June 2017 with an intensified schema of two sessions per week in the first month and then two sessions every 15 days. Within a year he was able to reduce the corticoid dose from 20 mg per day to 20 mg on alternate days and 2 years after starting ECP shows improvement of kidney function, describes feeling less tired in everyday activities and is now able to carry out mild exercises. In January 2020 ECP was suspended not to burden a low cardiac function.

**Figure 2.**
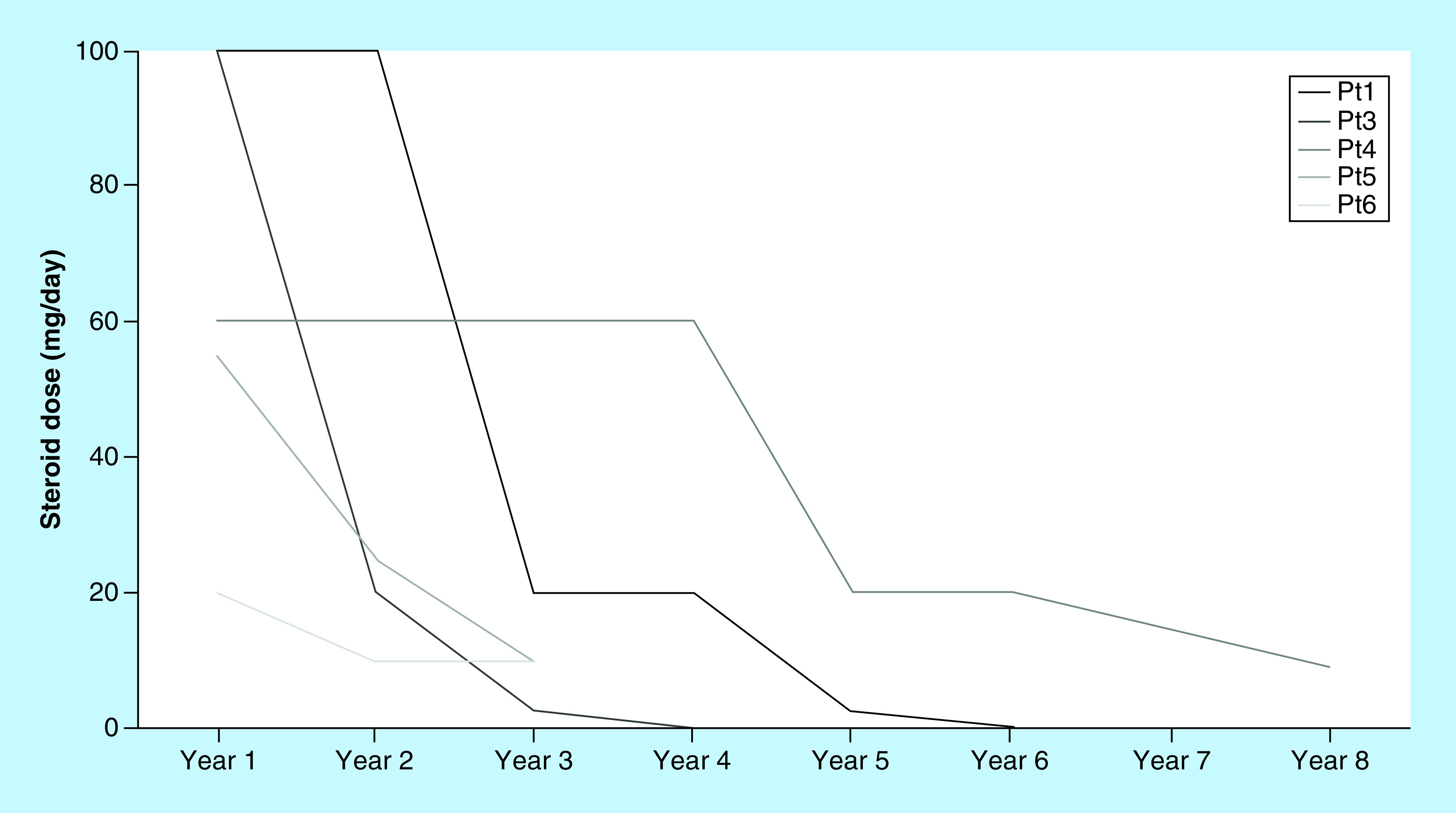
Reduction of the prednisolone scheme, in mg/day, evaluated per year under extracorporeal photopheresis. Patient two not shown due to nonprednisolone containing scheme. Pt: Patient.

All patients reported improvement of symptoms and were able to reduce steroid dose by the end of ECP (Figure 2). Pt2 had no initial response to glucocorticoids, but was still able to reduce the CsA dose from 400 to 150 mg/day.

### Immune monitoring

**Figure 3.**
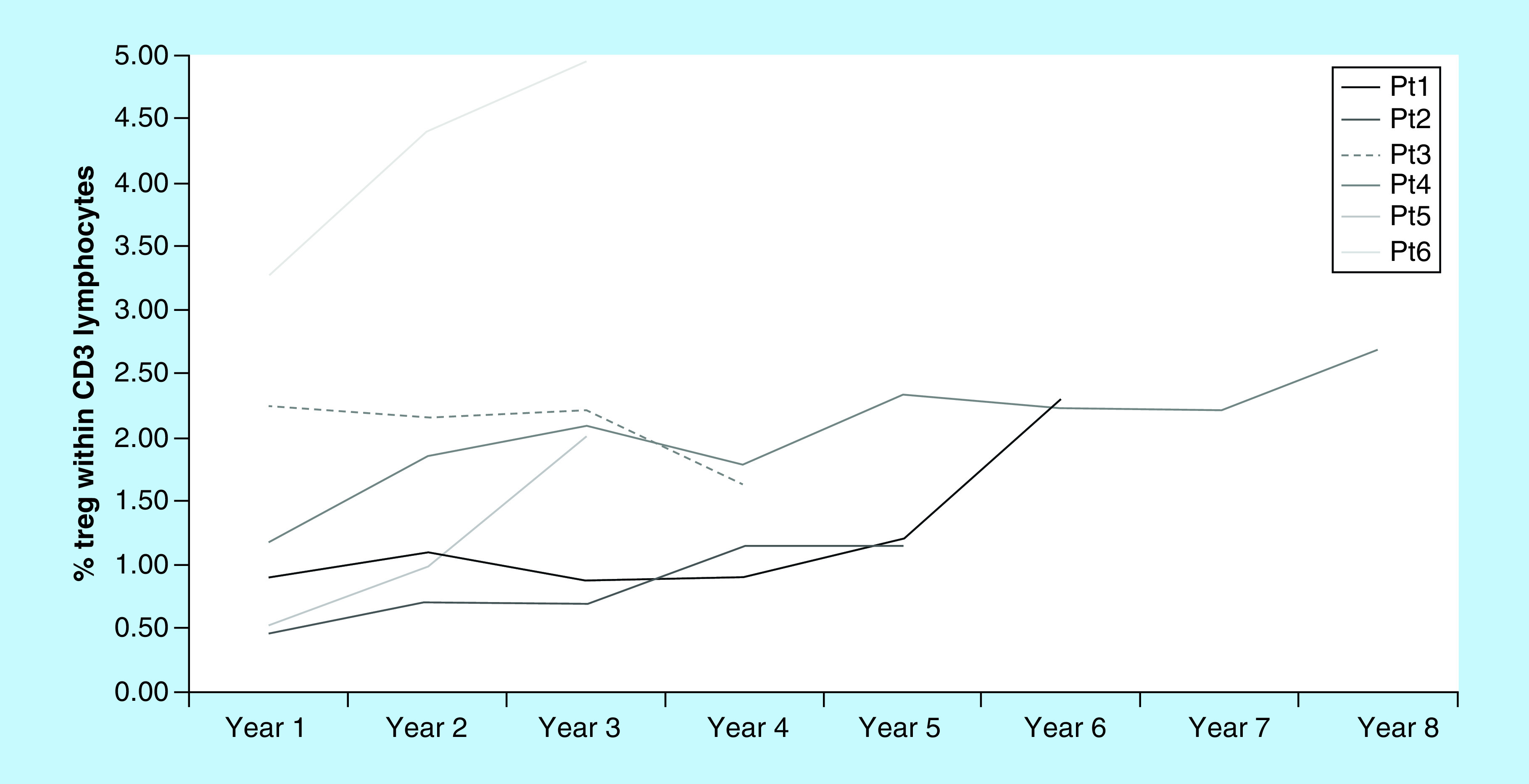
Distribution of Treg in percentage, mean value per year of extracorporeal photopheresis treatment for each patient. All responding patients had a significant increase in %Tregs comparing the first to the last year of treatment (p < 0.001). Patient three, who did not respond to extracorporeal photopheresis, had not (p = 0.19). Pt: Patient.

A blood sample was collected from each patient before the first day of treatment and sent to the flow cytometry lab, with a total of 370 samples analyzed. An increase in both the Treg percentage and the Th/Tc ratio was observed with time undergoing ECP. This steady increase was most noticeable when data was assessed per years of treatment (Figure 3).

With exception of Pt3, who was considered as nonresponder to ECP, the increase in the Treg percentage within the CD3^+^ T-cell population was statistically significant. Comparing values from the first and last year of ECP treatment, a significant increase in the %Treg was found in all responding patients (p < 0.001), but not in nonresponder Pt3 (p = 0.19). When a linear mixed model was applied, we observed a statistically significant positive trend in %Treg variation with time was observed (p < 0.001).

**Figure 4.**
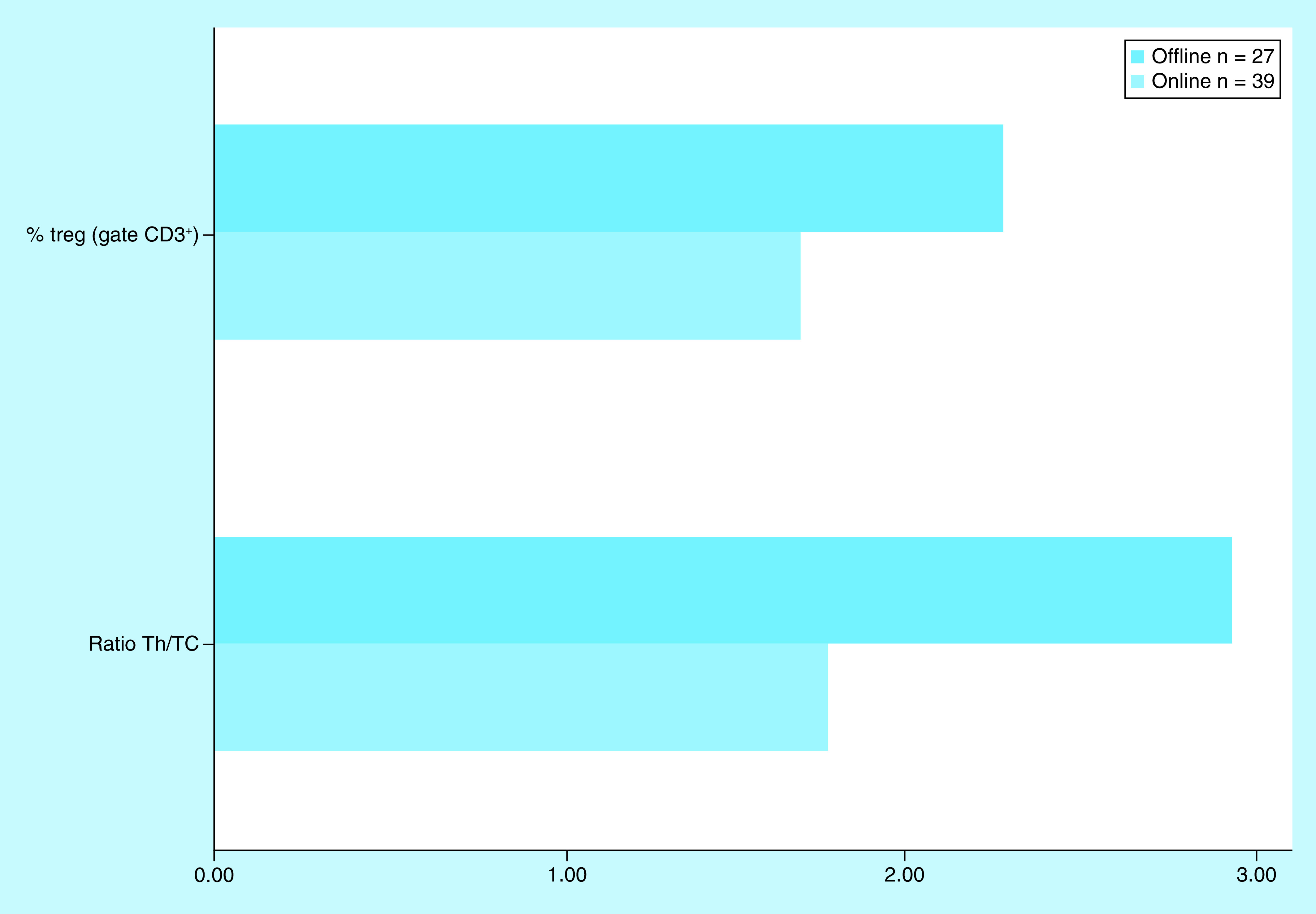
Offline extracorporeal photopheresis protocol increases the average circulating regulatory T cell and T helper/cytotoxic T cells ratio, when compared with the online protocol in patient four, p < 0.01. Th/Tc: T helper/cytotoxic T cell; Treg: Regulatory T cell.

Pt4 had 39 online immune evaluations versus 27 offline, so it was possible to compare the circulating Treg and Th/Tc ratio under each protocol (Figure 4). For both parameters a significant difference was found with the Students’ *t-*test (p < 0.01).

## Discussion

Despite the long-time clinical experience treating cGvHD patients with ECP, dating back to 1993 with apparent benefit, this technique is still not considered as a therapeutic option by some centers [17–19]. Available data relies mostly on small case series, with few clinical trials directly comparing ECP to other alternatives [11,20–22]. Although response rates vary among studies, improvement of symptoms are generally reported, always associated with an extremely low incidence of adverse reactions. When comparing with immunosuppressive therapy, ECP appears to be cost effective [23].

In all our six patients, ECP was well tolerated with no major AEs or signs of systemic immunosuppression reported in up to 8 years of treatment. Pt3, who had several complications other than cGvHD, was considered a nonresponder, but was still able to taper glucocorticoid therapy. The other long term ECP patients reduced or discontinued glucocorticoids and had improvement of symptoms, with one (Pt2) having no cGvHD. The decrease in the corticosteroid dose and clinical evaluation of our patients reinforces that there is a place for ECP in the management of cGvHD, at least in a well selected subset of patients. The interaction between ECP and immunosuppressive therapy must be addressed in future studies, if the full potential of both treatments is to be obtained.

The biological mechanisms by which ECP controls the action of reactive T-cell clones are not yet fully understood, but it seems to rely on the generation of regulatory cells [8]. Pt3 (considered a nonresponder) showed no significant variation in the percentage of circulating Treg. In the other five patients, a significant increase in the Treg percentage was observed (p < 0.001) with time and number of ECP sessions performed, although interpatient values vary. The helper to cytotoxic circulating T-cell ratio also increased in time, yet it appears to be less sensitive than the former. Other regulatory populations are also likely to be involved and should be explored in future studies [24].

In Pt4, it was possible to compare data from both the online and the offline protocols, with the later having significantly higher values in both parameters. Both Pt5 and Pt6 only performed the offline ECP protocol and showed a quick increase in the circulating Treg. Whether the differences found in Pt4 and the rapid response of Pts5 and 6 were due exclusively to the protocol cannot be determined with the available data. Further studies, comparing both protocols in larger series, should address this issue in the future. The difference in incubation time with 8-MOP may affect the effectiveness of treatment, as demonstrated *in vitro* [25].

While expression of Foxp3 is the classical hallmark of Treg, its value varies among studies and even within a given population at different time points. The quantification of Foxp3 expression requires intracellular staining and is phosphorylation-dependent, making the technique less suitable for routine analysis and more vulnerable to interanalyst variability. Although Treg are CD4^+^CD25^+^, this phenotype alone is neither sufficient nor adequate. The addition of CD127 allows for the identification of Treg, with a high correlation with CD4^+^ CD25^+^+Foxp3^+^ cells and regulatory activity [26,27]. We had previously validated this protocol comparing to Foxp3 staining, with overlapping results (data not published).

In a 14-patient series, Rubegni *et al.* also describe an increase in CD4^+^ CD25^+^ T lymphocytes with ECP and hypothesize a central role in the cascade of immunological events [28].

Denney *et al.* in a series of 32 cGvHD patients treated with ECP, reported an increase in circulating Treg but no correlation with cGvHD improvement [29]. In a large prospective trial of 83 patients, Gandelman *et al.* reported an improvement in NIH criteria, but no association with circulating Tregs [30]. In both studies, patients were followed up to 12 months, which may justify the different findings. As discussed here, we found the increase in circulating Treg most noticeable when data was assessed per years of treatment, accompanying a favorable response to treatment. A flow cytometry laboratory is common in centers performing HSCT and/or cellular therapy, ECP in particular, making circulating Treg evaluation a parameter suitable to monitor such patients.

The long follow-up time of patients allowed us to obtain a considerable amount of biological specimens with consistent results, however, we are aware that the small number of studied patients limits data extrapolation.

## Conclusion

ECP is a safe procedure, with low AEs and no increased relapse or immunosuppression reported. It is effective, ameliorating the symptoms of cGvHD and allowing the taper or withdrawal, of steroid therapy while maintaining the graft antitumor activity. ECP is also a feasible procedure, only requiring a venous access, nevertheless a well-equipped specialized aphaeresis center with a well-trained team is mandatory. Patients report feeling satisfied, which leads to a high treatment compliance. In responding patients an increase in circulating Tregs was observed but the usefulness of this parameter as a biomarker must be addressed in larger studies.

We consider ECP to be a personalized form of cellular therapy, allowing for the intensity and frequency of sessions to be adjusted to each patient. For all these reasons, we believe that ECP is a reliable therapy and should in fact be used more often.

## Future perspective

Considering the increasing recommendation for ECP in the treatment of cGvHD, due to both positive feedback from the patients and a very low incidence of AEs, it is expectable that this form of therapy becomes more frequent in a near future. The lack of large studies, with some inconsistent results across small, single center reports and the lack of reproducible and easily measured biomarkers to access patient response to treatment are issues to be address in the near future. It is our believe that once established which cohort of patients are more likely to benefit from ECP and the best tools to monitor such patients, ECP will assume an important role in the treatment of GvHD patients.

**Executive summary**

- Chronic graft-versus-host disease is a major late complication following hematopoietic stem cell transplant, with immune competent cells from the donor targeting patient’s tissues, in a manner similar to some autoimmune diseases.
- After initial response to steroid treatment many patients become steroid-refractory, with no consensual follow-up treatment.
- Extracorporeal photopheresis (ECP) is a form of cellular therapy, based on promoting tolerance rather than immunosuppression.
- Tregs are a cell population capable of reverting inflammatory responses, believed to main one of the major effectors of ECP induced tolerance.
- Response to ECP treatment is usually accompanied by an increase in circulating Treg, most noticeable when ECP is continued for long periods.
- In response to ECP, chronic graft-versus-host disease patients show improvement of symptoms and are able to reduce the dose of steroid, with an extremely low rate of adverse effects.
