# Corrigendum

**DOI:** 10.2144/fsoa-2020-0107c

**Published:** 2024-05-16

A Short Communication by authors Sérgio Machado Lopes, Susana Roncon, Ana Catarina Pinho, Filipa Bordalo, Luís Antunes, Fernando Campilho, António Campos & Altamiro Costa-Pereira was published in the September 2020 issue of *Future Science OA* 6(8), FSO623 (2020). The financial and competing interests disclosure has now been updated as follows:

‘This article was supported by National Funds through FCT – Fundação para a Ciência e a Tecnologia, I.P., within CINTESIS, R&D Unit (reference UIDB/4255/2020). The authors have no other relevant affiliations or financial involvement with any organization or entity with a financial interest in or financial conflict with the subject matter or materials discussed in the manuscript apart from those disclosed.

No writing assistance was utilized in the production of this manuscript.’

The editors and the authors of *Future Science OA* would like to sincerely apologize for any inconvenience or confusion this may have caused our readers.

